# Austrian Society of Gastroenterology and Hepatology (ÖGGH) consensus on primary biliary cholangitis

**DOI:** 10.1007/s00508-026-02798-6

**Published:** 2026-09-09

**Authors:** Emina Halilbasic, Elisabeth Tatscher, Elmar Aigner, Lukas Burghart, Ivo Graziadei, Stephanie Hametner-Schreil, Benedikt Silvester Hofer, Harald Hofer, Andreas Maieron, Mattias Mandorfer, Markus Peck-Radosavljevic, Benedikt Schaefer, Wolfgang Sieghart, Martin Stradner, Stefan Traussnigg, Martin Wagner, Heinz Zoller, Peter Fickert, Michael Trauner

**Affiliations:** 1 Division of Gastroenterology and Hepatology, Department of Medicine III, Medical University of Vienna, Vienna, Austria; 2 Division of Gastroenterology and Hepatology, Department of Internal Medicine, Medical University of Graz, Graz, Austria; 3 First Department of Medicine, University Clinic Salzburg, Paracelsus Medical University Salzburg, Salzburg, Austria; 4 Klinik Ottakring, Wiener Gesundheitsverbund, Vienna, Austria; 5 Department of Internal Medicine, Academic Teaching Hospital, Hall, Austria; 6 Department of Gastroenterology and Hepatology, Ordensklinikum Linz Barmherzige Schwestern, Linz, Austria; 7 Department of Internal Medicine I, Gastroenterology and Hepatology, Rheumatology, Endocrinology and Diabetology, Klinikum Wels-Grieskirchen, Wels, Austria; 8 Clinical Research Institute for Inflammation Medicine, Johannes Kepler University, Linz, Austria; 9 Department of Internal Medicine 2, Gastroenterology & Hepatology, University Hospital, St. Pölten, Austria; 10 Karl Landsteiner University of Health Sciences, Krems, Austria; 11 Innere Medizin und Gastroenterologie (IMuG), Klinikum Klagenfurt, Klagenfurt am Wörthersee, Austria; 12 Department of Internal Medicine I, Gastroenterology, Hepatology, Endocrinology and Metabolism, Medical University of Innsbruck, Innsbruck, Austria; 13 Imed19-Privat, Private Clinical Research Center, Vienna, Austria; 14 Division of Rheumatology and Immunology, Department of Internal Medicine, Medical University of Graz, Graz, Austria; 15 Dr Brunner & Dr Traussnigg – Fachärzte für Innere Medizin OG, Internisten am Elterleinplatz, Vienna, Austria

**Keywords:** PBC, Ursodeoxycholic acid, Elafibranor, Seladelpar, Liver transplantation

## Abstract

This consensus document of the Austrian Society of Gastroenterology and Hepatology (ÖGGH) is intended to provide practical guidance for the management of individuals with primary biliary cholangitis (PBC).

PBC is a chronic inflammatory, autoimmune-mediated disease of the intrahepatic bile ducts that can lead to fibrosis and ultimately cirrhosis. Middle-aged women are significantly more frequently affected than men. The pathogenesis is currently not fully understood. Based on the presence of disease-specific autoantibodies, it is classified as an autoimmune liver disease, although a combination of genetic predisposition and environmental factors contribute to disease development and progression.

The diagnosis of PBC is based on a cholestatic enzyme pattern together with the presence of anti-mitochondrial antibodies (AMA) or PBC-specific anti-nuclear antibodies (sp100, gp210). A liver biopsy is rarely required to establish the diagnosis; exceptions are the suspicion of a PBC autoimmune hepatitis (AIH) variant syndrome or the absence of the abovementioned antibodies.

The therapeutic goal is to reduce cholestatic injury thereby preventing disease progression and to reduce symptoms. Approximately 60–70% of patients achieve clinical and biochemical remission with first-line treatment, i.e., ursodeoxycholic acid (UDCA). Recently, the therapeutic paradigm has shifted from achieving certain predefined response criteria to a normalization of alkaline phosphatase (ALP) together with a low-normal bilirubin level, as the latter was linked to improved outcomes in some subgroups. For patients who do not sufficiently respond to UDCA, the newly approved peroxisome proliferator-activated receptor (PPAR) agonists elafibranor and seladelpar, as well as bezafibrate (*off-label* use), should be used as a combination treatment with UDCA. In patients with decompensated cirrhosis, liver transplantation has been associated with good long-term outcomes, albeit disease recurrence occurs in up to 50% by 15 years.

## Statements and recommendations

### Epidemiology, natural history and diagnosis of PBC

- Primary biliary cholangitis (PBC) is a rare autoimmune cholestatic liver disease predominantly affecting women between 40 and 70 years of age. The female-to-male ratio has decreased in recent decades.
- The disease can progress without clinical symptoms for years; however, fatigue and pruritus are highly prevalent and represent the most burdensome symptoms, substantially impairing health-related quality of life (HrQoL) independent of disease stage.
- The diagnosis of PBC is established when at least two of the following are present: **(A1)**
  - Persistently elevated parameters of cholestasis, in particular alkaline phosphatase (ALP), in the absence of biliary obstruction on imaging (e.g., abdominal ultrasound or magnetic resonance cholangiopancreatography, MRCP).
  - PBC-specific autoantibodies, e.g., anti-mitochondrial antibodies (AMA) or PBC-specific anti-nuclear antibodies (ANA), i.e. gp210 and sp100.
  - Characteristic histology showing chronic granulomatous, nonsuppurative and destructive cholangitis of interlobular and septal bile ducts, with lymphocytic infiltrates and bile duct loss in advanced stages.
- Particularly in patients with positive PBC-specific antibodies, polyclonal IgM elevation, isolated gamma-glutamyl transferase (GGT) elevation, and other indicative findings, such as hypercholesterolemia or symptoms including fatigue, pruritus, upper abdominal discomfort, sicca syndrome, or arthralgia may raise clinical suspicion of PBC, although not part of the standard diagnostic criteria.
- Liver biopsy is not routinely required for diagnosis, if PBC-specific autoantibodies and typical cholestatic biochemistry are present. **(B1)**
- Liver biopsy is recommended when:
  - PBC-specific autoantibodies are absent despite biochemical cholestasis with exclusion of biliary obstruction. **(C1)**
  - There is suspicion of PBC-autoimmune hepatitis (AIH) variant syndrome or a co-existing liver disease (e.g., metabolic dysfunction-associated steatotic liver disease (MASLD)) which would affect patient management. **(C1)**
- In individuals with PBC-specific autoantibodies and normal ALP, liver biopsy should not be routinely performed solely to establish a diagnosis of PBC; however, when GGT is elevated and alternative causes (e.g. MASLD) have been excluded, liver biopsy for establishing a diagnosis of PBC can be considered in selected cases. **(D2)**
- In individuals with PBC-specific autoantibodies and normal liver biochemistry, periodic biochemical reassessment for the development of liver disease can be considered every 1–2 years as a small proportion of these patients will develop PBC. **(B2)**
- Genetic testing for inherited cholestatic disorders can be considered to support the diagnostic process in patients lacking PBC-specific autoantibodies and if liver histology is not diagnostic of PBC. **(C2)**
- Disproportionate elevations of ALT to ALP should prompt consideration of an PBC-AIH variant syndrome or the suspicion of another concomitant liver disorder. **(B1)**
- The Paris criteria requiring at least two of the following: (1) ALT > 5× ULN, (2) IgG > 2× ULN and/or positive anti-SMA, and (3) moderate-to-severe interface hepatitis on liver biopsy remain the so far best-validated and most commonly used tools for diagnosing PBC-AIH variant syndrome. **(C1)**
- At diagnosis and during follow-up of PBC, associated symptoms, particularly pruritus, fatigue, sicca syndrome, arthralgia and other common extrahepatic autoimmune conditions, should be systematically assessed. **(C1)**

### Risk stratification, monitoring and follow-up

- Accurate disease staging at baseline and during follow-up is recommended to identify patients at risk of progression and to guide individualized therapeutic strategies. **(A1)**
- Risk stratification in PBC should be based on a combination of clinical (age), biochemical (bilirubin, ALP, AST, albumin, platelet count), immunological (anti-gp-210), and (where available) histological parameters (advanced fibrosis, ductopenia, and interface hepatitis), as well as noninvasive tests (elastography and/or ELF test) and assessment of portal hypertension. **(B1)**
  - Younger age at diagnosis (< 45–50 years) should be considered a risk factor for poorer outcomes and reduced likelihood of biochemical response.
  - Male sex and the presence of anti-gp210 antibodies have also been associated with unfavorable prognosis, although the supporting evidence is less consistent.
  - Anti-centromere antibodies (ACA) may be associated with disease progression and portal hypertension.
- In PBC without cirrhosis or advanced fibrosis serum ALP and bilirubin levels are key prognostic markers:
  - ALP > 2 × ULN and/or bilirubin > 1 × ULN after 1 year of therapy are strongly associated with adverse outcomes.
  - Achieving normalization of ALP and low-normal bilirubin (≤ 0.6× ULN) is associated with improved survival.
- Clinical and laboratory follow-up should be performed at 3, 6, and 12 months during the first year after diagnosis. Thereafter, follow-up intervals should be individualized according to disease activity, therapeutic response, and, where appropriate, additional risk factors. **(B1)**
- Non-invasive fibrosis assessment is recommended at diagnosis and during follow-up for prognostic evaluation. **(B1)**
  - Liver stiffness measurement (LSM) using vibration-controlled transient elastography (VCTE) is recommended as a key noninvasive tool for baseline risk assessment and monitoring. **(B1)**
  - LSM should be used for risk stratification, with thresholds (< 8 kPa, 8–15 kPa, > 15 kPa) identifying low, intermediate, and high-risk groups. **(C1)**
  - A current LSM > 10 kPa defines compensated advanced chronic liver disease (cACLD) and identifies those at risk for hepatic decompensation, irrespective of biochemical response.
  - Longitudinal changes in LSM are prognostically relevant, with decreasing values associated with improved outcomes but the most recent value should guide clinical decision making.
  - The enhanced liver fibrosis (ELF) score can be used for risk stratification, with higher values indicating increased risk and values < 9.8 suggesting low risk of liver-related events. **(C2)**
- Periodic reassessment of LSM using VCTE is recommended. (**B1)**
  - Annual assessment should be considered in patients with LSM > 10 kPa, persistent symptoms (pruritus) and/or inadequate biochemical response. **(C1)**
  - Longer intervals (2–3 years) might be appropriate in asymptomatic or minimally symptomatic low-risk patients with stable disease and optimal or adequate biochemical response. **(C2)**
- Non-invasive criteria combining LSM and platelet count (i.e. applying the BAVENO VII CSPH rule-out criteria LSM < 15 kPa combined with a platelet count ≥ 150 G/L) should be used to identify patients at low risk of varices/decompensation, although caution is warranted due to limited validation in PBC. (**C1)**
- Clinically significant portal hypertension (CSPH) should be considered even in non-cirrhotic PBC patients and is associated with increased risk of decompensation and mortality. (**B1)**
- Regarding management of portal hypertension and its complications, the Billroth IV recommendations and subsequent updates can be applied; although PBC-specific evidence is limited. (**B2)**
- Cirrhosis, defined by an LSM > 15 kPa or histology, should be considered the principal risk factor for HCC in PBC. (**A1)**
- Risk factors for HCC include male sex, older age, inadequate biochemical response, and advanced disease stage. (**B2)**
- HCC surveillance should be performed according to current guidelines, with liver ultrasound every 6 months and optional serum alpha-fetoprotein (AFP) measurement, in patients with PBC and cirrhosis. **(B1)**
- Ultrasound surveillance can also be considered in patients with suspected advanced fibrosis on elastography (≥ 10 kPa) and/or an inadequate biochemical response to treatment. (**C2)**

### Pharmacological treatment of PBC

- Management of PBC should focus on preventing disease progression, improving transplant-free survival, optimizing symptom control and quality of life, and managing comorbidities. **(A1)**
- All patients with PBC, including those with compensated or decompensated cirrhosis, should be treated promptly after diagnosis with oral ursodeoxycholic acid (UDCA) at a dose range of 13–15 mg/kg/day. **(A1)**
- UDCA can be administered as a single daily dose or in divided doses depending on tolerability. **(C2)**
- In patients with suspected gastrointestinal intolerance, dose adjustment or gradual dose re-escalation should be considered. **(C1)**
- Response to UDCA treatment should be assessed using established biochemical criteria (e.g. Paris II, Toronto) as early as 6 months (particularly in patients at higher risk of disease progression) with formal evaluation no later than 12 months after treatment initiation. **(B1)**
- A complete biochemical response (normal ALP and bilirubin) or adequate biochemical response (e.g. Paris II, Toronto) should be considered an optimal or acceptable treatment target **(B1)**.
- In patients with an inadequate biochemical response to UDCA, adherence, correct dosing, PBC-AIH variant syndrome, and concomitant liver diseases should be systematically evaluated. **(B1)**
- UDCA should be continued even in patients with inadequate response, as it provides clinical benefits. **(B1)**
- Achieving normalization of ALP and bilirubin < 0.6 × ULN (deep response) may be the optimal treatment goal associated with improved clinical outcomes and reduced progression of liver disease, although this strategy requires further validation.
- Treatment targets in PBC should be individualized based on risk stratification; the benefits of ALP normalization appear to be most pronounced in younger patients and those with a LSM ≥ 10 kPa. **(C1)**
- In patients with inadequate response or intolerance to UDCA initiation of an add-on second-line treatment should be implemented, preferably after referral to expert care. **(B1)**.
- At present, PPAR agonists are recommended as add-on second-line treatment in patients with insufficient response or intolerance to UDCA. **(A1)**
  - The selective PPAR agonists, elafibranor (80 mg/day) and seladelpar (10 mg/day), are EMA-approved treatment options.
  - Bezafibrate (200–400 mg/day) is an *off-label* treatment alternative.
  - Currently, the lack of head-to-head data does not enable firm conclusions on comparative efficacy between drugs.
- PPAR agonists are not recommended in decompensated cirrhosis and should be used with caution in compensated cirrhosis (Child Pugh A and B) **(B1)**.
- Potential adverse effects of PPAR agonists, particularly hepatotoxicity, myopathy, and renal dysfunction, warrant careful monitoring and consideration, especially in light of the differing safety profiles of individual agonists. **(C1)**
- In cases of inadequate response to one PPAR agonist patients can be offered an alternative PPAR agonist. **(D2)**
- The combination of different PPAR agonists is not recommended. **(B1)**
- Where available, triple therapy combining a PPAR agonist, FXR agonists such as obeticholic acid (OCA), and UDCA could represent a treatment option for selected patients. **(D2)**
- In patients with PBC and features of interface hepatitis or persistently elevated transaminases despite anticholestatic therapy, add-on treatment with budesonide can be considered, provided cirrhosis, portal hypertension and MASH comorbidity have been excluded. **(B2)**
- In patients with PBC-AIH variant syndrome, combined treatment with UDCA and immunosuppressive therapy is recommended. **(B1)**

### Treatment of symptoms and extrahepatic manifestations

- Symptoms of PBC, particularly pruritus and fatigue, should be assessed at each visit using standardized tools, e.g., a numeric rating scale (NRS) for pruritus and treated if clinically significant. **(B1)**
- Patients with pruritus should be advised on general supportive measures, including skin care with emollients, avoidance of skin irritation, cooling strategies, and appropriate clothing. **(C1)**
- First-line treatment with UDCA should not be expected to improve pruritus.
- PPAR agonists currently available as add-on second line treatment have shown promising effects on pruritus.
- Bezafibrate may be considered (*off-label*) for the treatment of pruritus, particularly in patients not receiving other PPAR agonists. **(C2)**
- IBAT inhibitors can be considered in patients with moderate-to-severe pruritus. **(C2)**
- Cholestyramine can be used as an alternative first-line treatment in mild pruritus, ensuring appropriate timing relative to other medications. **(C2)**
- Rifampicin can be considered as an *off-label* second-line treatment of pruritus with close monitoring of liver function. **(C2)**
- Naltrexone or sertraline can be considered as further *off-label* options in refractory cases. **(C2)**
- Patients with refractory pruritus should be referred to specialized centers for consideration of nonpharmacological therapies (e.g. UVB therapy, extracorporeal liver support systems, external or nasobiliary drainage). **(C1)**
- Evaluation of fatigue should include screening for alternative conditions, such as anemia, hypothyroidism, metabolic disturbances, sleep disorders, and depression. **(B1)**
- Management should focus on treating underlying causes and contributing factors, as well as supportive and behavioral strategies. **(B1)**
- Currently, no pharmacological treatment can be recommended for fatigue in PBC as no therapies are formally approved. Selective PPAR agonists have demonstrated some efficacy on fatigue.
- Non-pharmacological interventions (e.g. physical activity, especially aerobic and resistance-based exercise, behavioral therapies) can be considered, although evidence remains limited. **(C2)**
- Comorbidities should be systematically assessed, as they impact prognosis, treatment tolerability, and quality of life. **(B1)**
- Patients with PBC should be considered at increased risk of osteoporosis and fractures and should undergo osteoporosis risk assessment and treatment according to current national clinical practice guidelines. **(B1)**
  - Fracture risk should be assessed (e.g. using FRAX) at diagnosis. **(B1)**
  - Bone mineral density assessment (DEXA) should be performed in patients with intermediate or high fracture risk. **(B1)**
  - Serum calcium, phosphate, vitamin D, and parathyroid hormone should be measured at diagnosis and monitored regularly. **(B1)**
- Prevention and management of osteoporosis in patients with PBC should follow established national clinical practice guidelines, including fall prevention, cessation of smoking, adequate calcium, vitamin D, and protein intake. Antiresorptive or osteoanabolic therapy should be considered in patients with moderate and initiated in those with high fracture risk. **(B1)**
- Testing for thyroid disease (TSH, thyroid stimulating hormone) is recommended **(B1).**
- Symptoms of sicca syndrome should be actively assessed in patients with PBC. **(B1)**
- Artificial tears and saliva substitutes should be used as first-line therapy for mild sicca syndrome. **(B1)**
- Referral to a rheumatologist should be considered for severe sicca syndrome, suspected Sjögren’s disease and arthralgia. **(C1)**
- Lipid profile should be assessed in all patients with PBC. **(B1)**
- Cardiovascular risk should be evaluated individually, as PBC-related hypercholesterolemia alone does not necessarily increase risk. **(B1)**
- Decision on initiating lipid lowering therapy should be based on the risk for the development of major cardiovascular events calculated by the appropriate scores (e.g. SCORE or SCORE-2). **(B2)**

### Liver transplantation in PBC

- Indications for liver transplantation in PBC should follow general criteria, while considering disease-specific features. **(A1)**
- Referral for liver transplantation assessment should be considered in PBC patients with:
  - Persistently elevated or progressively rising bilirubin (≥ 3 mg/dL) **(B1)**
  - Development of complications of cirrhosis **(B1)**
  - Severe pruritus refractory to pharmacotherapy **(B1)**
- Recurrent PBC should be recognized as a common long-term phenomenon affecting a substantial proportion of transplant recipients; younger age at diagnosis or transplantation, tacrolimus use, and post-transplant cholestasis should be considered risk factors for recurrence, and histology remains the gold standard for diagnosis when recurrence is suspected **(B1).**
- All patients undergoing liver transplantation for PBC should receive pre-emptive treatment with UDCA at 10–15 mg/kg/day, as it reduces the risk of recurrence, graft loss, and mortality. **(B1)**
- In histologically confirmed recurrent PBC, PPAR agonists can be considered; however, evidence supporting this strategy remains limited, and transplant-specific safety concerns including potential drug-drug interactions should be carefully evaluated. **(D2)**

### Pregnancy in PBC

- Pregnancy in women with well-controlled disease and without advanced fibrosis is not associated with adverse maternal or fetal outcome.
- Pregnant patients with PBC should be closely monitored by a multidisciplinary team, including obstetricians and hepatologists. **(C1)**
- Caution and close surveillance are recommended in patients with cirrhosis. **(C1)**
- Postpartum follow-up should be performed, as cholestatic flares can occur after delivery. **(C1)**
- UDCA should be continued during pregnancy, as it is considered safe and non-teratogenic at recommended doses. **(C1)**
- UDCA can be used throughout all trimesters and during breastfeeding. **(C2)**
- Pruritus during pregnancy should be actively managed, and specialist input should be considered. **(B1)**
- Cholestyramine or rifampicin (*off-label*) can be considered for treatment of pruritus if symptoms are not adequately controlled with UDCA. **(B2)**
- Fibrates (e.g. bezafibrate *off-label*) can be considered after the first trimester on a case-by-case basis, if potential benefits outweigh risks. **(C2)**
- Selective PPAR agonists (elafibranor, seladelpar) should not be used during pregnancy or breastfeeding, due to lack of safety data. **(C1)**

## Introduction

This consensus document of the Austrian Society of Gastroenterology and Hepatology (ÖGGH) is intended as a practical aid for the diagnosis and management of primary biliary cholangitis (PBC). Our aim is to enhance awareness of the disease, present the current state of scientific knowledge and to improve patient care.

The certainty in the evidence and strength of recommendations was determined in analogy to the grading of recommendations, assessment, development, and evaluations (GRADE) framework (https://dev-bestpractice.bmjgroup.com/info/us/toolkit/learn-ebm/what-is-grade/) [1], if applicable:

- Very low (D): the true effect is probably markedly different from the estimated effect./Any estimate of effect is very uncertain.
- Low (C): the true effect might be markedly different from the estimated effect./Further research is very likely to have an important impact on our confidence in the estimate of effect and is likely to change the estimate.
- Moderate (B): the authors believe that the true effect is probably close to the estimated effect./Further research is likely to have an important impact on our confidence in the estimate of effect and may change the estimate.
- High (A): the authors are confident that the true effect is similar to the estimated effect./Further research is very unlikely to change our confidence in the estimate of effect.

Strength of recommendation:

- Weak (2): indicates that engaging in a shared decision-making process is essential.
- Strong (1): suggests that it is not usually necessary to present both options.

## Epidemiology, natural history and diagnosis of PBC

### Epidemiology, symptoms and natural history of PBC

PBC is considered a rare disorder, its incidence ranges from 0.23–5.31 per 100,000 individuals per year and its prevalence from 1.91–40.2 per 100,000, with considerable variation across different geographic regions [2–4]. PBC primarily affects women between 40 and 70 years of age, with a peak incidence at the fifth to sixth decade of life. PBC is extremely rare before the age of 25 years, although exceptional cases of adolescent onset have been reported [5,6]. PBC is traditionally considered to be predominantly a female disease; however, PBC in males appears to be more prevalent than previously assumed with an increasing global incidence [7].

The etiology of the disease remains incompletely understood; however, a complex interplay between genetic susceptibility and environmental exposures is thought to play a key role. In line with its autoimmune nature, approximately 95% of patients exhibit anti-mitochondrial antibodies (AMA) directed against mitochondrial antigens, while around 30% present with PBC-specific anti-nuclear antibodies (ANA) [8]. Immune-mediated bile duct injury leads to cholestasis with subsequent accumulation of toxic bile constituents, which in turn promotes hepatic damage, fibrosis, and, if untreated or inadequately treated, may ultimately progress to advanced chronic liver disease (ACLD)/cirrhosis.

Autoimmune conditions are frequently observed in this context, with Sjögren’s disease (in 19–31%), autoimmune thyroiditis (in up to 25%), and rheumatoid arthritis (in 6%) being the most commonly reported [9,10]. Due to a presumably increased prevalence of celiac disease in PBC, screening has been recommended according to some scientific societies [8]; however, a 2025 meta-analysis including 15,006 patients with PBC found a biopsy-confirmed celiac disease prevalence of 1.5–1.7%, comparable to the approximately 1% prevalence in the general population, suggesting no substantially increased risk among patients with PBC [11]. Testing for celiac disease should therefore be reserved for patients with clinical suspicion or relevant symptoms (such as fatigue and/or iron deficiency).

Bile duct injury in PBC progresses silently, and thus, patients can initially remain asymptomatic for several years. Health-related quality of life (HRQoL) in PBC can be hampered by symptoms such as fatigue, pruritus, unspecific upper abdominal pain, cognitive issues (“brain fog”), sicca syndrome, and arthralgia [12]. Pruritus and fatigue are the most burdensome symptoms in PBC, affecting up to 70% and 80% of patients, respectively, significantly impairing daily, mental, and physical functioning regardless of disease severity. Despite its high prevalence, pruritus is frequently underreported and inadequately documented by clinicians, underscoring an important gap in patient care [13]. Several assessment tools are available: The PBC-40 evaluates overall symptom burden, while pruritus-specific tools include the 5‑D itch scale, visual analog scale (VAS), and numerical rating scale (NRS). Fatigue can be assessed using instruments such as the fatigue severity score (FSS) and fatigue impact scale (FIS). While valuable in clinical trials, the complexity and time requirements limit the feasibility of these tools in routine clinical care [14].

Pruritus occurs in approximately 55–89% of patients during the disease course, with 25–53% experiencing moderate to severe symptoms, most commonly affecting the limbs, trunk, scalp, and groins. It contributes to sleep disturbance, fatigue, depression, and social isolation [15–18].

Fatigue is a common symptom of PBC with a major impact on HRQoL. According to a recent meta-analysis, the pooled prevalence of fatigue in PBC is high, with an overall estimate of approximately 63%, ranging from 20% up to 94% depending on study population and test used [14]. Due to its subjective and multifactorial nature, fatigue remains difficult to quantify. Its pathophysiology in PBC is poorly understood and is likely influenced by factors such as pruritus, depressive symptoms, daytime somnolence, and autonomic dysfunction [14]. Fatigue should not be equated with simple tiredness. Pathological fatigue refers to a state of physical and/or cognitive exhaustion, ranging from persistent weariness to profound exhaustion, that is disproportionate to prior activity, unrelieved by rest and associated with functional impairment [14]. Fatigue can be conceptualized as central, peripheral, or mixed, with central fatigue marked by cognitive (“brain fog”) and motivational impairment and peripheral fatigue by reduced muscle strength and endurance [14–19]. Patients might not spontaneously report fatigue, presumably because they do not recognize this symptom as disease-related. Furthermore, time-limited clinical encounters and a focus on biochemical markers often result in these symptoms being overlooked [13–20]. Fatigue in PBC is typically a persistent symptom that does not improve over time [14]. The only long-term study on fatigue in PBC found that fatigue remained consistent after 4 years of monitoring and was not influenced by changes in disease severity or biochemical response to treatment with ursodeoxycholic acid (UDCA) [21]. This suggests that fatigue is a chronic and enduring symptom for many PBC patients, significantly adding to their overall symptom load. Clinicians should be encouraged to adopt a proactive, structured approach, using targeted questions besides spontaneously reported symptoms [22]. This enables identification of disease manifestations that might otherwise remain hidden, particularly in patients who understate or struggle to describe their symptoms.

PBC is increasingly diagnosed at older ages, and thus, in patients in whom the clinical course of the disease is less severe and in whom the disease may have previously been missed. Together with therapeutic improvements, this has led to a marked reduction in liver-related 10-year mortality, from 35% in the 1970s to 6% in the 2000s [23,24] and in the proportion of PBC as transplantation indication among liver transplant recipients, from 12% in the 1980s to 2% in the 2020s [25]. Current international registry studies indicate a 10-year survival rate of nearly 80% for patients treated with UDCA; however, it is crucial to acknowledge the risks of untreated PBC. Historical population-based cohorts (UK and USA) have shown that untreated PBC patients had an average survival of 9–16 years from presentation, with 25% developing liver failure during this period [26,27].

### Diagnosis of PBC

PBC is nowadays most commonly identified through routine laboratory testing in asymptomatic individuals, with persistent elevation of cholestatic liver enzymes, particularly alkaline phosphatase (ALP), representing a key biochemical hallmark of the disease. Elevation of gamma-glutamyl transferase (GGT) can also cause suspicion for PBC and trigger testing for elevated ALP and autoantibodies (see below). Other indicative findings may include laboratory abnormalities such as an isolated and polyclonal increase in immunoglobulin M (IgM) levels or hypercholesterolemia [28].

**Fig. 1:**
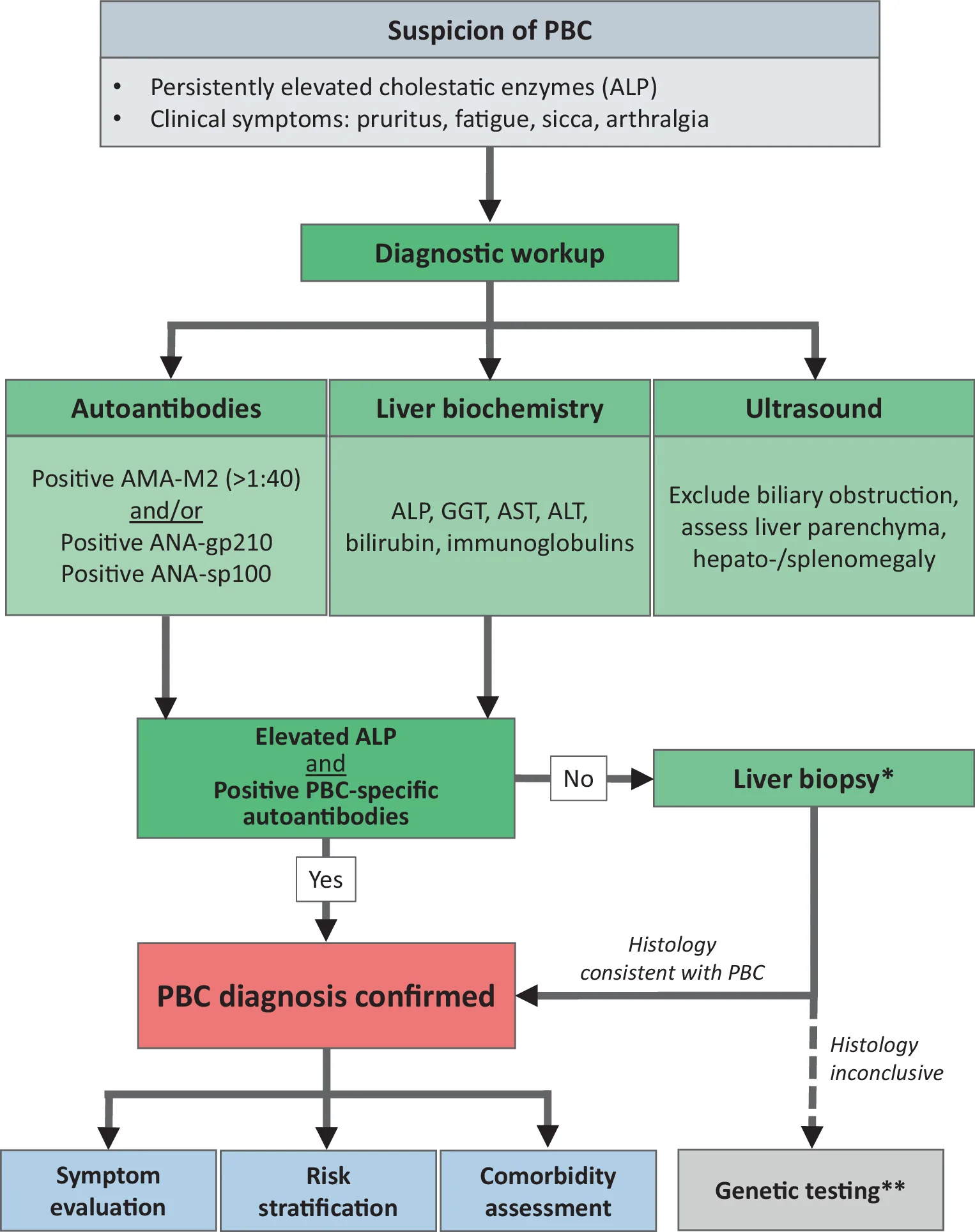
Diagnostic approach in patients with clinical suspicion of PBC. *Liver biopsy is also recommended when a PBC-AIH variant syndrome, other cholangiopathies (e.g. small duct PSC) or a coexisting liver disease (e.g. MASLD) is suspected. **Genetic testing for inherited cholestatic disorders (e.g. mutations in the transporter genes ATP8B1, ABCB11 and ABCB4) may be considered in patients lacking PBC-specific autoantibodies when liver histology is compatible with, but not diagnostic of PBC. *ABCB11* ATP-binding cassette subfamily B member 11 (also bile salt export pump BSEP); *ABCB4* ATP-binding cassette subfamily B member 4 (also multidrug resistance protein 3 MDR3); *AIH* autoimmune hepatitis; *ALP*, alkaline phosphatase; *ALT* alanine aminotransferase; *AMA* anti-mitochondrial antibodies; *ANA* anti-nuclear antibodies; *AST* aspartate aminotransferase; *ATP8B1* ATPase phospholipid transporting 8B1 (also familial intrahepatic cholestasis 1, FIC1); *GGT* gamma-glutamyl transferase; *MASLD* metabolic dysfunction-associated steatotic liver disease; *PBC* primary biliary cholangitis; *PSC* primary sclerosing cholangitis

The definitive diagnosis of PBC is based on a combination of laboratory findings and, if required, histopathology (Fig. 1).

The diagnosis of PBC is established when at least two of the following three criteria are met:

- Chronically (> 6 months) elevated parameters of cholestasis, in particular ALP. Isolated persistent GGT elevation may represent the earliest biochemical stage of PBC and support the diagnosis in the presence of PBC-related autoantibodies after exclusion of other causes [29,30].
- Detection of a disease-specific autoantibody profile consisting of anti-mitochondrial antibodies M2 (AMA-M2, present in approximately 90–95% of patients) or specific anti-nuclear antibodies (ANA) directed against nuclear dots (sp100) or the nuclear membrane (gp210) using indirect immunofluorescence testing or immunoassays.
- Characteristic histology showing chronic granulomatous, nonsuppurative, and destructive cholangitis of interlobular and septal bile ducts, with lymphocytic infiltrates and bile duct loss in advanced stages.

Abdominal ultrasound should be performed as the first-line imaging modality to exclude obstructive cholestasis in patients with suspicion of PBC. Magnetic resonance cholangiopancreatography (MRCP) (when conditions such as primary or secondary sclerosing cholangitis (PSC or SSC) or IgG4-related cholangitis are suspected) or endoscopic ultrasound (EUS) (in suspected distal biliary obstruction) can be performed alternatively or in addition to abdominal ultrasound. There are no ultrasound findings specific for PBC; however, abdominal, particularly hilar, lymphadenopathy is detectable in 87–97% of patients [31–34]. An elevation of alanine aminotransferase (ALT) disproportionate to ALP can reflect increased inflammatory disease activity but should also prompt consideration of a PBC-autoimmune hepatitis (AIH) variant syndrome or the presence of another concomitant liver disorder.

### Methodology and quality requirements of serological diagnostics

AMA positivity is found in 95% of PBC patients [35,36]. It can be detected by indirect immunofluorescence on triple rodent tissue sections (stomach, liver, kidney) or on HEp2 cells (cytoplasmic reticular pattern, AC-21). The AMA-M2 refers to a subtype of AMA directed against the M2 antigen complex located in the inner mitochondrial membrane, which can be detected by ELISA or dot blot. In a meta-analysis, AMA detected with indirect immunofluorescence yielded a sensitivity of 85% and a specificity of 98%. The sensitivity of AMA-M2 by ELISA was 83% with a specificity of 95% [37]. One study used an immunoblot to detect AMA-M2 (sensitivity 91%, specificity 100%) [37]. If AMA are absent and there is still clinical suspicion of PBC, antibodies against other antigens should be tested [31–38]. These include ANA with multiple nuclear dots pattern (AC-6) against the sp100 antigen and punctate nuclear envelope pattern (AC-12) against gp210. Anti-sp100 and anti-gp210 antibodies are highly specific and present in over 30% of PBC patients negative for AMA; the presence of anti-gp210 correlates with a poor prognosis [38–42]. Anti-centromere antibodies (ACA) have a low specificity for PBC but testing can be considered [31–43] as ACA positivity represents a risk factor for development of portal hypertension [39]. Anti-kelch-like 12 (anti-KL12) and anti-hexokinase 1 (anti-HK1) antibodies have been proposed to be present in approximately 40% of AMA negative patients with PBC [44,45]; however, tests for these antibodies are not yet widely available.

The clinical significance of isolated AMA-M2 positivity in asymptomatic patients without laboratory evidence of cholestasis remains unclear as these antibodies can also be detected in approximately 0.5% of the healthy population. In a study involving 229 individuals without a confirmed diagnosis of PBC who were followed for up to 7 years, the 5‑year incidence of PBC was 16%, suggesting that only 1 in 6 patients with positive AMA and normal ALP levels would develop PBC within 5 years [46]. Another recent study including 1018 individuals found that, over a median follow-up of 6.9 years, only 7.9% of AMA positive individuals developed PBC, with a higher risk observed in those with higher AMA titers [47]. Small patient cohorts in which liver biopsy was performed despite normal ALP levels and positive AMA showed high rates (40–80%) of histological features consistent with PBC [30–48,49]. As the disease can develop over time, clinical practice guidelines recommended that AMA-positive individuals undergo biochemical reassessment for liver disease every 1–2 years until the age of 65 years; however, data supporting this recommendation are limited [10–50].

### Role of liver biopsy

Liver histology is generally not required at initial diagnosis but should be performed when the diagnosis is uncertain (e.g., in cases of absence of disease-specific autoantibodies) or when an additional liver disease (e.g., metabolic dysfunction-associated steatotic liver disease, MASLD or a PBC-AIH variant syndrome) is reasonably likely and would affect patient management (Fig. 1). Histological diagnosis of PBC depends on biopsy size and the number of evaluable portal tracts, with detection of cholangitis and bile duct damage increasing with tract number [51]. Findings should be interpreted cautiously, as characteristic lesions can be absent, especially at early disease stages [8]. A biopsy can also be considered when a rapid progressive ductopenic variant is suspected in patients with elevated direct bilirubin but no signs of cirrhosis. This distinct subgroup of often younger patients with PBC (up to 5–10%) is characterized by severe cholestasis and marked ductopenia in the absence of significant fibrosis or cirrhosis, with faster disease progression and higher risk of treatment failure, need for liver transplantation and higher mortality rates [52,53].

In AMA-positive individuals with normal ALP, liver biopsy should not be performed to establish a diagnosis of PBC, as the limited available evidence does not indicate that the benefits outweigh the risks [54,55]; however, when GGT is elevated and alternative causes (e.g., MASLD) have been excluded, liver biopsy can be considered in selected cases [56].

Patients with PBC presenting with AST and/or ALT > 5 times the upper limit of normal (ULN), IgG > 2 × ULN, or with positivity to autoantibodies, e.g., ANA (excluding PBC-specific gp210 and sp100), smooth muscle antibodies (SMA), liver kidney microsomal antibody type 1 (LKM-1), liver cytosol type 1 antibody (LC-1), soluble liver antigen/liver pancreas antibody (SLA/LP), should undergo liver biopsy. Although mild to moderate interface hepatitis can be found as a hepatocellular response to cholestatic liver injury in PBC (previously termed “active” PBC), its severe form is a hallmark of AIH. The coexistence of features of PBC and AIH is referred to as the PBC-AIH variant (previously known as “overlap”) syndrome and occurs in up to 20% of PBC patients. Histology alone is insufficient to establish a definitive diagnosis. Although autoantibody profiles can provide useful information, they should not be used in isolation to ensure a reliable and consistent assessment [57]. In the absence of universally accepted diagnostic criteria for PBC-AIH variant syndrome, the Paris criteria (1998), the best-validated and guideline-endorsed system, can be used requiring at least two of the following: (1) ALT > 5× ULN, (2) IgG > 2× ULN and/or positive anti-SMA, and (3) moderate-to-severe interface hepatitis on liver biopsy [58]. Zhang et al. proposed new diagnostic criteria based on the revised AIH scoring system, modifying the International Autoimmune Hepatitis Group classification by incorporating selected histological features of both AIH and PBC together with adjusted biochemical and immunological parameters; these “Zhang criteria” (with optimal diagnostic performance at a score of ≥ 21) appear to have higher sensitivity than the Paris criteria, which could underestimate the prevalence of PBC-AIH variant syndrome [59,60].

Differentiating PBC-AIH variant syndrome from difficult to treat PBC (partial/null response to UDCA) remains challenging and referral to tertiary centers is recommended. AIH-associated serological or histological features can occur without altering the diagnosis, prognosis or treatment, underscoring the need for cautious interpretation to avoid overtreatment [57–61]; however, some patients may benefit from combination therapy consisting of corticosteroids/immunosuppressants and UDCA [60]. Thus, it is crucial to identify those who are most likely to benefit while avoiding unnecessary immunosuppression.

Given that AIH and PBC features can occur simultaneously or sequentially and that prognostically relevant AIH flares could be missed, lifelong close monitoring is mandatory in all patients with PBC [10–61].

In patients with symptoms and signs indicative of PBC; but without PBC-specific autoantibodies and with liver histology that is compatible with, but not diagnostic for PBC, genetic testing for inherited cholestatic disorders (e.g., mutations in *ATP8B1, ABCB11*, and *ABCB4*) can be considered [10].

## Risk stratification, monitoring and follow-up

**Fig. 2:**
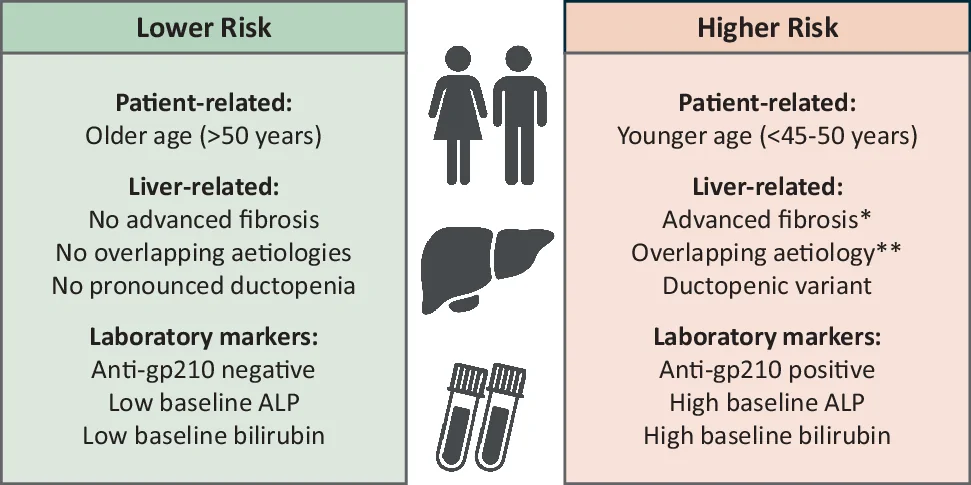
Risk stratification in PBC. Accurate risk stratification at baseline and during follow-up is essential for identifying patients at increased risk of disease progression and guiding individualized therapeutic strategies. Risk assessment integrates age, routine biochemical tests, immunological profiles, serum fibrosis markers, imaging findings (e.g. liver ultrasound), liver stiffness measurement and histopathology (if available). *LSM determined by e.g. VCTE (> 10 kPa); **AIH, MASLD. Modified from [62]. *ALP* alkaline phosphatase

Accurate disease staging at baseline and throughout follow-up is fundamental to individualized patient care, enabling the identification of individuals at increased risk of disease progression and guiding decisions on the need for tailored therapeutic interventions. Risk stratification in PBC is based on multiple parameters, including patient demographics, routine biochemical tests, immunological profiles, serum markers of fibrosis, imaging findings (such as liver ultrasound to detect cirrhosis), liver and spleen stiffness measurements (LSM and SSM), histopathological features, and the evaluation of portal hypertension (Fig. 2; [10]).

**Fig. 3:**
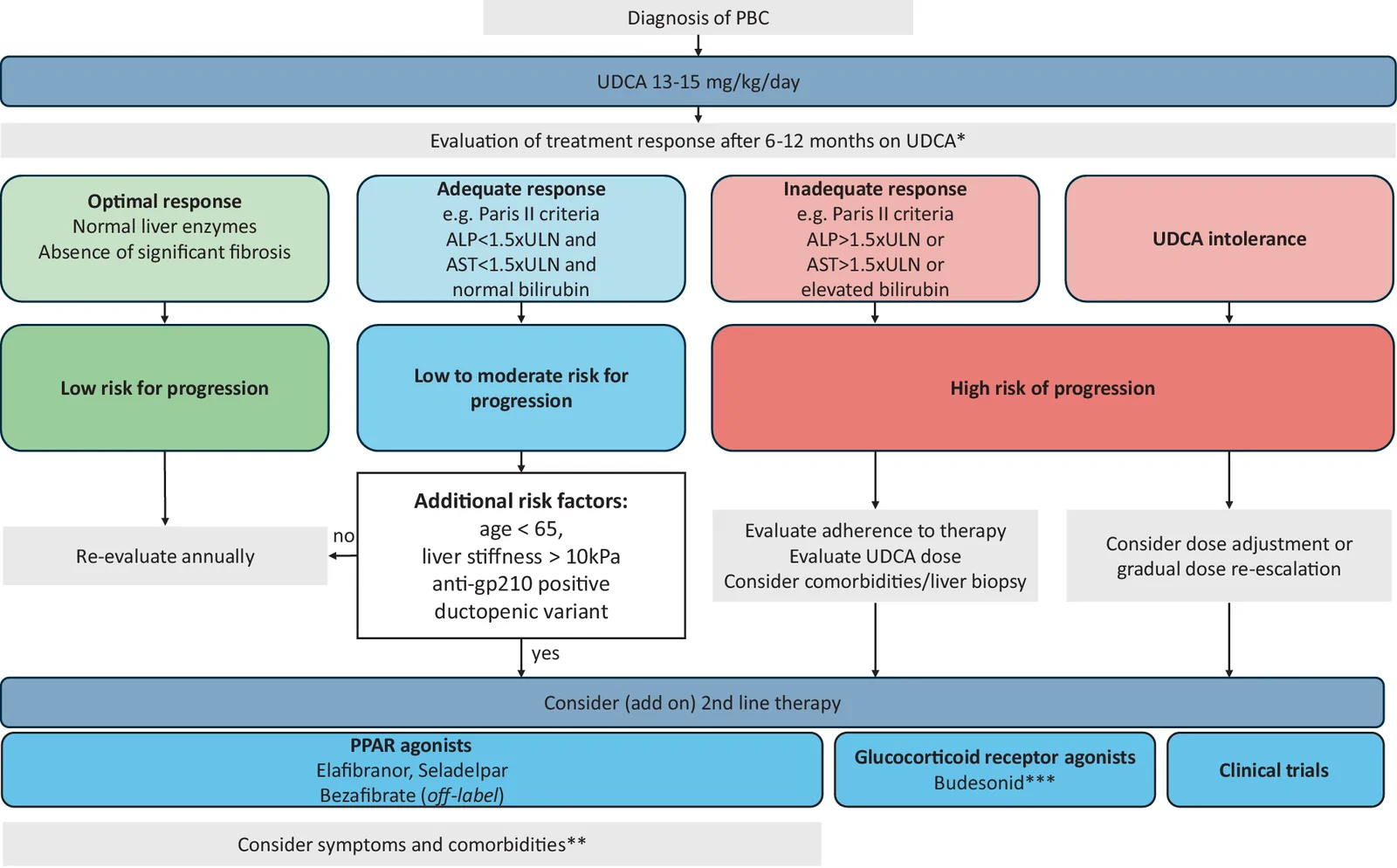
Risk-stratified therapeutic approach in patients with PBC. *Absence of response by different criteria (e.g. Paris II or Toronto) after 6 months: low likelihood of response after 12 months [63]; **The choice of combination or second-line therapy may take into account the presence of symptoms, comorbidities and concomitant medication. ***In patients with PBC and features of interface hepatitis or persistently elevated transaminases despite anticholestatic therapy, provided cirrhosis and portal hypertension have been excluded. Modified from [64]. *ALP* alkaline phosphatase; *AST* aspartate aminotransferase; *UDCA* ursodeoxycholic acid; *ULN* upper limit of normal; *PBC* Primary biliary cholangitis; *PPAR* Peroxisome proliferator-activated receptor

Clinical and laboratory follow-up should be performed at 3, 6, and 12 months during the first year after diagnosis. Thereafter, follow-up intervals should be individualized according to disease activity, therapeutic response and, where appropriate, additional risk factors [31–61, 65].

Younger age at initial diagnosis is associated with a poorer prognosis in PBC [66]. Large PBC cohorts (UK and global) found that patients diagnosed before 45–50 years of age had a lower likelihood of meeting UDCA response criteria, experienced more symptoms like itching and fatigue and are more likely to undergo liver transplantation or death compared to those diagnosed at older age [67,68]. Among patients aged ≥ 55 years at diagnosis, mortality rates are comparable to those observed in an age-matched and sex-matched general population [69].

The impact of sex as a risk factor is debated. No clear link between male sex and transplant-free survival was found in the global PBC cohort; however, men are at higher risk of developing hepatocellular carcinoma (HCC) [67–70], more frequently diagnosed at an advanced stage of disease, less likely to respond to UDCA treatment than women and face an overall increased risk of complications [67–71]. The worse outcomes observed in men are likely due to delayed diagnosis and consequently more advanced disease at presentation, rather than sex as a modifier of the disease course itself [68].

In a limited number of predominantly retrospective studies, the presence of the most commonly reported symptoms, fatigue and pruritus, has been associated with a poorer response to first-line therapy, more rapid disease progression and reduced overall survival [27–61, 65–72,73]; however, the lack of a standardized, globally accepted approach to assessing symptom severity limits their prognostic utility in clinical practice [54].

Biochemical markers have been central to risk stratification in PBC for decades, with ALP and bilirubin remaining key prognostic indicators. Lower levels of ALP have been associated with reduced mortality and longer transplant-free survival; conversely, ALP > 2 × ULN at baseline and at 1 year of follow-up, together with bilirubin > 1 × ULN at 1 year, are strongly predictive of adverse outcomes, including liver-related death and need for liver transplantation [74]. Also, patients with highly elevated baseline ALP levels, particularly those with ALP > 4–5× ULN, have a lower likelihood of achieving a biochemical response after 1 year of UDCA monotherapy [1–61, 63, 65–75].

Multicenter PBC cohort studies have shown that patients achieving a low-normal bilirubin under standard therapy (≤ 0.6 × ULN, along with normalization of ALP) have significantly higher survival rates compared with patients whose bilirubin remains within the normal range but above this threshold [76].

Data from the Global PBC Study Group demonstrate that elevated serum GGT, particularly levels > 3.2 × ULN at 12 months, are independently associated with an increased risk of liver transplantation or liver-related death, even in patients with ALP < 1.5 × ULN [77]; however, interpretation should consider potential confounders such as MASLD and alcohol use.

The presence of PBC-specific ANA, particularly anti-gp210, has been associated with more advanced disease and poorer outcomes, including lower response rates to UDCA, increased liver-related mortality, and reduced transplant-free survival [41–61, 65–78], while ACAs have been linked to disease progression and an increased risk of portal hypertension [39].

Inadequate biochemical response to first-line therapy is one of the strongest risk factors for disease progression. Prognostic scores from the UK-PBC Research Group (UK-PBC score, https://www.uk-pbc.com/resources/tools/riskcalculator/) and the Global PBC Study Group (GLOBE score, https://www.globalpbc.com/globe) estimate individual risk of disease progression and complications after initiation of UDCA treatment [79,80]. These scores are based on large multicenter cohorts and incorporate disease stage (albumin, bilirubin, platelets) and age at diagnosis. A GLOBE score > 0.30 after 1 year of standard treatment predicts significantly reduced transplant-free survival in PBC patients [80].

Although liver biopsy is rarely required for the diagnosis of PBC, it provides valuable information on disease progression, prognosis, and treatment response, and can support risk stratification and therapeutic decision-making [54]. Histological features, particularly fibrosis stage, ductopenia (> 50% bile duct loss), deposition of copper-associated protein granules, and the severity of lymphocytic interface activity, are associated with clinical outcomes and can help predict progression to cirrhosis and liver failure [53–61, 65–83]. Given that current guidelines discourage the employment of liver biopsy for diagnosis, coupled with the increasing availability of LSM by vibration-controlled transient elastography (VCTE) for significant fibrosis and ACLD/cirrhosis, the importance of histological analysis for evaluating the prognosis of PBC has decreased, as it cannot easily be repeated over time.

In current practice, non-invasive tools for assessing fibrosis should be used at diagnosis and during follow-up for prognostic assessment and monitoring [28–61, 65–74].

The enhanced liver fibrosis (ELF) score reliably reflects fibrosis stage and prognosis in PBC, with higher baseline values associated with shorter event-free survival and a threefold increase in complication risk per point increase [84,85], while an ELF score < 9.8 demonstrates a high negative predictive value (~95%) for liver-related events [86].

With respect to LSM, VCTE, the most extensively studied modality in PBC, is recommended for staging and monitoring [54]. Growing evidence supports the use of LSM for risk stratification in PBC with respect to the outcomes of hepatic decompensation, liver transplantation, or death [87]. As such, LSM thresholds of > 9.6 kPa at baseline and progression > 2.1 kPa/year (ΔLSM) were associated with a markedly increased risk of adverse outcomes including liver failure, the need for a liver transplantation, or death [88]. In a large international multicenter cohort of 3985 PBC patients, LSM was independently associated with poor clinical outcomes, with cut-offs of < 8 kPa and > 15 kPa effectively stratifying patients into low, intermediate and high-risk groups for poor clinical outcomes [87]. In an international multicenter cohort of 1793 PBC patients, the most recent LSM was the strongest predictor of hepatic decompensation, with values > 10 kPa markedly increasing risk, irrespective of prior LSM trajectory or biochemical response, supporting the use of current LSM for simplified risk stratification [89].

Besides baseline assessment, periodic reassessment of LSM seems essential, as even patients with seemingly stable biochemical profiles may still require adjustments to the treatment. Longitudinal assessment of VCTE-LSM was shown to provide important prognostic information in PBC, as reductions in LSM, regardless of cause, baseline values, or other prognostic factors, are associated with a lower risk of serious clinical events [90].

Although evidence on optimal intervals for elastography follow-up is limited, annual reassessment appears reasonable for patients with liver stiffness above 10 kPa and/or inadequate biochemical response to enable early detection of disease progression and timely initiation of interventions, such as HCC surveillance or variceal screening.

In asymptomatic or only minimally symptomatic patients and lower risk of disease progression (i.e., < 10 kPa, stable disease, and complete biochemical response), longer intervals of 2–3 years between elastography assessments may be appropriate.

In PBC, clinically significant portal hypertension (CSPH) can occur even before the development of cirrhosis and is associated with more advanced histological changes, such as fibrous septa formation and nodular regenerative hyperplasia [91,92]. The occurrence of CSPH is linked to an increased risk of hepatic decompensation and mortality and is thought to be initially predominantly presinusoidal, with an additional sinusoidal component emerging as the disease progresses [93]. Up to 27% of patients have been reported to present with ACLD at diagnosis, and more than half of these exhibit features of CSPH [94].

CSPH is defined by a hepatic venous pressure gradient (HVPG) ≥ 10 mm Hg or by clinical signs of PH (i.e., varices or portosystemic collaterals). Although HVPG is the reference standard, it does not capture the presinusoidal component of portal hypertension in PBC, while non-invasive tests may be sufficiently accurate for clinical use. Elastography-based criteria (Baveno VI/VII) are well-validated to rule out high-risk varices in viral, alcohol-related and non-obese steatotic liver disease and have been shown to be useful in PBC patients, as LSM < 15 kPa combined with a normal platelet count (≥ 150 G/L) identified patients at a negligible 10-year risk of hepatic decompensation or death [94–96]. Nevertheless, the performance of elastography-based criteria to actually rule out CSPH in PBC is less certain, with limited validation data and higher false negative rates reported especially in patients with ALP levels higher than 1.5 × ULN [97,98]. Risk stratification in PBC may be further improved by SSM and SSM ≤ 40 kPa may help to rule out high-risk varices [99]. Regarding management of portal hypertension and its complications, the Billroth IV recommendations and subsequent updates can be applied; although PBC-specific evidence is limited [100].

Cirrhosis is the strongest risk factor for HCC development in PBC [101]. Additional risk factors include inadequate biochemical response (including failure to meet Paris I criteria), older age, male sex, concomitant liver diseases and thrombocytopenia, with 10-year HCC incidence < 5% in biochemical responders but > 30% in male nonresponders to UDCA [70]. Surveillance of HCC should be performed according to current guidelines, with liver ultrasound every 6 months and optional serum alpha-fetoprotein (AFP) measurement, in patients with PBC and cirrhosis [102]. Ultrasound surveillance can also be considered in patients with suspected advanced fibrosis on elastography (≥ 10 kPa) and/or an inadequate biochemical response to treatment.

## Pharmacological treatment of PBC

In the absence of curative treatment, management of PBC focuses on controlling disease progression, improving transplant-free survival, optimizing HrQoL through symptom control and managing comorbidities; therefore, all patients, including those with ACLD/cirrhosis, should receive treatment promptly after diagnosis. (Fig. 3) 

### Ursodeoxycholic acid

Oral ursodeoxycholic acid (UDCA) at 13–15 mg/kg/day is recommended as the standard initial therapy in all patients with PBC. UDCA can be given as a single oral daily dose (preferably at bedtime [103]) or divided doses if tolerability is an issue. Based on three double-blind randomized control trials (total of 548 PBC patients) UDCA treatment showed a one third reduction in the risk of death or need for liver transplantation for patients with moderate to severe PBC [104]. UDCA is generally well-tolerated, with only a few minor side effects reported when given at the recommended dosage, such as a possible weight increase within the first year, some hair loss, and infrequently, diarrhea and bloating. Intolerance to UDCA has been reported in 3–5% of patients. It should be evaluated whether this represents true intolerance or, more commonly, gastrointestinal side effects (e.g. increased stool frequency and abdominal discomfort) which can often be managed by dose reduction followed by gradual re-escalation [31]. If tolerated, UDCA treatment should be continued lifelong in PBC. Notably, UDCA has no proven effect on patient reported outcomes (PROs) such as pruritus or fatigue [50].

The therapeutic response to UDCA based on biochemical parameters such as ALP, AST, and bilirubin should be assessed 6 but no later than 12 months after initiation of treatment [31] and should continue during the course of the disease. In patients with suspected insufficient response to UDCA, which is observed in about 25–40% of patients, it must be ensured that the diagnostic criteria for PBC are met, that there is no evidence of PBC-AIH variant syndrome or a concomitant liver disease (e.g., MASLD). It must also be ensured that UDCA is taken as prescribed (treatment adherence) and that the administered dose is appropriate, as real-world data show that UDCA doses below the recommended 13 mg/kg/day can be observed in nearly one third of patients [31–61, 65–105,106]. Nonadherence has been reported in up to 11% of patients, with younger age and male sex identified as factors associated with reduced treatment compliance [107].

Adequate response to UDCA can be assessed using several criteria including simple dichotomous criteria such as Barcelona, Paris I and II, Rotterdam, Rochester I and II, Toronto, and mathematical models such as the GLOBE and the UK-PBC scores ([79,80], Table 1). Most criteria evaluated the response based on laboratory (and clinical) parameters 12 months after treatment initiation, with the exceptions of Rochester I (6 months) and Toronto (24 months) [13–61, 65–108]. Therefore, the evaluation, and if necessary, adapting alternative treatment was so far recommended 12 months after the UDCA initiation [10–61, 65–109].

**Table 1:** Evaluation of treatment response using dichotomous criteria

Criteria	Months after starting UDCA	ALP threshold	Bilirubin threshold	AST threshold	Other thresholds
Paris I	12	< 3× ULN	< ULN	< 2× ULN	–
Paris II	12	≤ 1.5× ULN	< ULN	≤ 1.5× ULN	–
POISE	12	≤ 1.67× ULN or < 15% decrease	< ULN	–	–
Barcelona	12	< ULN > 40% decrease	–	–	–
Toronto	24	≤ 1.67× ULN	–	–	–
Rotterdam	12	–	< ULN	–	And/or albumin > LLN

*ALP* alkaline phosphatase; *AST* aspartate aminotransferase; *LLN* lower limit of normal; *UDCA* ursodeoxycholic acid; *ULN* upper limit of normal.

**Table 2:** Add-on second line treatment options in PBC patients with inadequate response to UDCA

	PPARδ seladelpar (approved) (Hirschfield NEJM 2024 [110])	PPARα/δ elafibranor (approved) (Kowdley NEJM 2023 [111])	Pan-PPAR bezafibrate (off label) (Corpechot NEJM 2018 [112])	GR (PXR) budesonide (off label) (Hirschfield J Hep 2021 [113])	FXR obeticholic acid (currently no approval) (Nevens NEJM 2016 [114])
Biochemical response	62%* (after 12 months)	51%* (after 12 months)	67%** (after 24 months)	43%* (after 36 months)	47%* (after 12 months)
Response over placebo	42%	47%	31%	20%	37%
ALP normalization	25%	15%	31%	35%	7%
Baseline ALP^+^	314.6 ± 123.0	321.3 ± 121.9	244 (211–308)	262 [IQR 286]	316–326
cACLD (%)	14%	34%	19%	0%	20%
Effect on symptoms	↓ Pruritus + fatigue	↓ Pruritus + fatigue	↓ Pruritus	–	–
Adverse events	Gastrointestinal Headache	Gastrointestinal Headache Myalgia (CK ↑) Cholelithiasis	Gastrointestinal Myalgia (CK ↑) Creatinine ↑ Cholelithiasis Increase in ALT	Gastrointestinal Hypertension Muscle spasms Arthralgia Osteopenia/porosis Peripheral edema Cataract Weight increase	Pruritus
CKD adjustment	No dosage adjustment required	No dosage adjustment required	Contraindicated if GFR < 60 ml/min	–	–
Women of childbearing age	Avoid during pregnancy (no embryo-fetal toxicity in preclinical models)	Contraindicated during pregnancy (embryo-fetal toxicity in preclinical models)	Contraindicated during pregnancy	–	–

*cACLD* compensated advanced chronic liver disease; *ALP* alkaline phosphatase; *ALT* alanine aminotransferase;* CK* creatinine kinase; *CKD* chronic kidney disease; *GFR* glomerular filtration rate; *GR* glucocorticoid receptor; *IQR* interquartile range; *PPAR* peroxisome proliferator-activated receptor; *PXR* pregnane X receptor

*ALP < 1.67× ULN with at least 15% reduction from baseline and normal bilirubin

**Normal bilirubin, ALP, AST, ALT, albumin and INR

^+^Mean values and range of baseline ALP according to respective publication. Please note that no head-to-head studies are available, and comparisons are limited by differences in baseline ALP levels and the proportion of patients with cACLD

Studies from real-world evidence suggest that it is possible to predict the outcome of UDCA treatment sooner, such as within the first 6 months of treatment [75], or even prior to treatment initiation [115,116]. As such, failure to achieve a biochemical response according to established response criteria (e.g. Paris II or Toronto criteria) at 6 months is associated with a low likelihood of achieving response at 12 months [63]. Furthermore, an ALP level > 1.9× ULN after 6 months of UDCA treatment was associated with a 90% probability of an inadequate treatment response at later time points [75].

With effective second-line therapies now available for patients with an inadequate response to UDCA, timely evaluation integrating clinical judgment, biochemical markers, and other relevant clinical variables is essential to identify patients most likely to benefit from treatment escalation. Reassessment as early as 6 months may be especially reasonable in patients with features indicating a higher risk of disease progression (Fig. 2; [13]).

While an “adequate response” to UDCA has traditionally been defined using the binary biochemical criteria mentioned above (e.g. Toronto, Paris II), emerging evidence supports the concept of a “deep response,” characterized by normalization of ALP and bilirubin < 0.6× ULN as the “optimal” goal to be achieved, as it has been shown to be associated with improved long-term outcomes [76–109, 115–117]. In a large retrospective cohort study of patients with PBC and adequate biochemical response to UDCA followed up over 15 years, normalization of ALP levels was associated with significant absolute and relative gains in complication-free survival at 10 years, particularly in patients with advanced fibrosis (LSM ≥ 10 kPa) and/or age ≤ 62 years [118]. Persistent ALP elevation, even within accepted response thresholds, may thus reflect ongoing disease activity [119] and selected higher risk patients (as mentioned above) may derive additional benefit from deeper biochemical normalization [118]; however, this approach cannot yet be generalized to all patients with PBC and further evidence is required to balance potential benefits against the risks of overtreatment. Results from ongoing open-label extension studies may clarify whether achieving a deep response translates into a survival benefit and supports its adoption as a new clinical standard [54].

In patients with an inadequate response to UDCA therapy and/or ACLD, the diagnosis, treatment initiation, and follow-up should be re-evaluated by gastroenterologists and hepatologists experienced in PBC management, and if UDCA monotherapy remains insufficient, an add-on second-line therapy should be initiated based on the patient’s individual risk of disease progression and symptom burden [31]. The use of UDCA should be continued at the same dose, as it reduces the risk of adverse liver outcomes, even in patients with inadequate response [120].

### Add-on second line treatment in PBC patients with inadequate response to UDCA or UDCA intolerance

Early identification of patients with an inadequate response to UDCA monotherapy, who are at increased risk of disease progression, is essential, and add-on second-line therapy should be initiated based on the individual risk of progression and symptom burden. In PBC patients with risk factors for disease progression (age < 65 years and/or advanced fibrosis ≥ 10 kPa), a combination therapy may be considered even in cases of only mildly elevated ALP [31–61, 65–109, 115–118]. Although normalization of ALP (“deep response”) has been proposed as a treatment target, current phase 3 trials have excluded patients with only mildly elevated ALP (≤ 1.5–1.67× ULN), and the efficacy of such an approach in this population remains uncertain so far.

### Selective PPAR agonists elafibranor and seladelpar (licensed)

Elafibranor is a selective PPAR‑α and PPAR‑δ agonist (predominant target isoform based on EC_50_: PPAR‑α [121]) that received conditional European Medicines Agency (EMA) approval in 2024 for second-line therapy in PBC. In the multinational phase 3 ELATIVE trial, 161 participants received either 80 mg of elafibranor once daily or placebo (2:1) for 12 months [111]. The primary endpoint (ALP < 1.67× ULN, ≥ 15% reduction from baseline ALP, and normal bilirubin) was achieved by 51% of patients in the elafibranor group versus 4% in the placebo group. ALP normalization occurred in 15% of elafibranor-treated patients compared with 0% in the placebo arm [111]. Overall, elafibranor was well-tolerated with the most common adverse events being self-limiting gastrointestinal symptoms (abdominal pain, nausea, vomiting, diarrhea) [111]. Discontinuation rates due to adverse events were similar for elafibranor (10%) and placebo (9%) [111]. Creatine kinase elevation and myalgia were slightly more common with elafibranor, especially in individuals with concomitant statin use [111] (Tab. 2).

Seladelpar, a selective PPAR‑δ agonist, received a conditional marketing authorization in February 2025. In the phase 3 RESPONSE trial, 193 patients received 10 mg seladelpar once daily or placebo (2:1) for 12 months [110]. The primary endpoint, defined as ALP < 1.67× ULN with ≥ 15% reduction and normal bilirubin, was achieved by 62% of seladelpar-treated patients versus 20% in the placebo group [110]. Normalization of ALP occurred in 25% of patients on seladelpar versus 0% on placebo. Seladelpar also resulted in a statistically significant improvement in pruritus compared with placebo. Seladelpar showed a safety profile comparable to placebo, with fewer discontinuations (3% vs. 5%) and no treatment-related serious adverse events. The most common adverse events were mild gastrointestinal symptoms (abdominal pain, nausea and abdominal distention) and headache [110]. Liver and muscle safety were similar between groups [110] (Tab. 2).

An interim analysis from 337 patients on seladelpar up to 2 years (ASSURE study), demonstrated durable improvements in cholestatic biomarkers and pruritus, and revealed no new safety concerns up to 2 years of treatment [122].

Patients with decompensated liver disease were excluded from the phase 3 studies, although 14% (RESPONSE, 18 patients classified as cirrhosis) and 34% (ELATIVE, 35 patients with LSM > 10 kPa or bridging fibrosis (or both) or cirrhosis), respectively, had cACLD [110,111]. While acknowledging the relatively small number of patients with cirrhosis, seladelpar was associated with higher response rates and greater reductions in ALP than placebo at 12 months in patients with cACLD, with sustained improvements observed for up to 18–24 months in the open-label extension (ASSURE) [123]. Bilirubin remained stable, no treatment-related serious adverse events were reported, and only a small number of patients developed hepatic decompensation events [123].

Elafibranor and seladelpar appear to have more favorable safety and tolerability profiles than fibrates [121]. Long-term extension data suggest favorable safety for both agents, while further real-world and controlled studies are needed to better define their safety profiles [121]. Elafibranor has been linked to occasional ALT and creatine kinase elevations, whereas seladelpar has not shown increases in liver or muscle biomarkers at approved doses [121]. For both drugs, no negative effects on renal function were observed, although patients with chronic kidney disease or impaired renal function were excluded from the phase 3 trials [110,111]. An increased fracture risk was noted with elafibranor (6% of cases) and seladelpar (4% of cases) compared with 0% in the respective placebo groups [110,111]. Therefore, consistent with standard care in PBC, monitoring of bone density should be performed.

Drug-drug interactions with oral contraceptives have been reported for elafibranor; furthermore, elafibranor is contraindicated during pregnancy due to adverse pregnancy outcome without direct evidence of teratogenic potential in preclinical models (fetal loss, malformations, stillbirths and/or perinatal deaths). Seladelpar is also not recommended during pregnancy; however, no embryo-fetal toxicity was observed in preclinical studies (EU-SMPC, EU Summary of Product Characteristics).

### Fibrates (*off label*)

The pan-PPAR agonist bezafibrate (predominant target isoform based on EC_50_: PPAR‑α [121]) and PPAR‑α agonist fenofibrate have been used for many years as cost-effective second-line options in PBC, although they are not formally approved for this indication (*off-label* use) [124]. Activation of PPAR‑α upregulates genes involved in bile acid and lipid metabolism, while downregulating genes associated with immune-mediated processes [125]. Both PPAR‑γ and PPAR‑δ exert additional anti-inflammatory and anti-fibrotic effects [126,127]. Several small or retrospective studies have demonstrated beneficial effects of both fenofibrate and bezafibrate in combination with UDCA [128–130]. In a large Japanese cohort including nearly 4000 patients, add-on bezafibrate was associated with improved transplant-free survival [131]. The randomized, placebo-controlled phase 3 BEZURSO trial enrolled 100 patients with PBC and incomplete UDCA response who received 400 mg bezafibrate or placebo (1:1), in combination with UDCA, for 24 months [112]. The primary endpoint of complete biochemical response (normalization of ALP, bilirubin, aminotransferases, albumin, and prothrombin time) was achieved in 31% of bezafibrate-treated patients versus 0% in the placebo group [112] (Tab. 2). Bezafibrate was associated with a reduction in liver stiffness by 15%, whereas placebo was associated with an increase of LSM by 22% in one study [112]. In a cohort study with a 5-year follow-up, the addition of bezafibrate in PBC with an incomplete response to UDCA significantly improved liver biochemistry and histological parameters, including a marked reduction in fibrosis and inflammatory scores [132]. Despite beneficial effects on biochemical markers and liver stiffness, fibrates should be used with caution. Careful patient selection and close monitoring for adverse effects are recommended [133]. Recognized risks include renal dysfunction, limiting their use in chronic kidney disease, myalgia, especially with concomitant statin therapy, and transaminase elevations via CYP2C9 inhibition [134,135]. In the BEZURSO trial, 20% of bezafibrate-treated patients reported myalgia (vs. 10% with placebo); one case of rhabdomyolysis occurred in a patient receiving a statin [112]. In the Japanese cohorts, hepatotoxicity occurred in 6% of patients and required steroid therapy in a subset of cases [112, 131–134]. A recent real-world Dutch nationwide study showed a 1-year discontinuation rate of almost 25% [136]. This was mainly due to side effects (i.e. gastrointestinal symptoms and myalgia) [136].

Small retrospective studies suggest that triple therapy with UDCA, obeticholic acid (OCA), and fibrates may improve cholestasis markers and symptoms in difficult to treat patients with insufficient response to second-line treatment [137,138]. A phase 2 trial presented at the 2024 EASL Congress reported normalization of all liver biochemical markers in 66.7% of patients after 12 weeks of triple therapy (OCA, UDCA, bezafibrate), although results remain preliminary and the study is ongoing [139]. Given the withdrawal of OCA approval for the EU in 2024, the future role of this triple therapy is currently uncertain.

Due to limited evidence and potential safety concerns, PPAR agonists are generally not recommended in decompensated liver disease and should be used cautiously in patients with compensated cirrhosis.

### Immunosuppressants

Budesonide is a synthetic corticosteroid with extensive first-pass hepatic metabolism and therefore fewer systemic side effects than prednisolone; however, its pharmacokinetics deteriorate with advancing liver disease and it can result in adverse outcomes (e.g., portal vein thrombosis) in patients with cirrhosis and portal hypertension [140] where it is contraindicated. In patients with PBC and interface hepatitis, budesonide in combination with UDCA has demonstrated efficacy in improving liver biochemistry and histology. Although elevated transaminases in PBC may reflect hepatocellular injury due to cholestasis rather than active inflammation, the combination of budesonide and UDCA may exert a biliary protective effect. Several studies have shown that budesonide added to UDCA in early-stage PBC can reduce serum ALP levels and improve histological features; however, results in non-cirrhotic PBC have been inconsistent, with reports of both regression and progression of liver fibrosis. The phase 3 trial was underpowered, limiting definitive conclusions [113] (Tab. 2). Particular attention is required when assessing treatment response in patients with PBC-AIH variant syndrome, which is associated with a poorer prognosis. Early initiation and timely optimization of treatment are therefore critical. Patients with moderate to severe interface hepatitis benefit most from combined treatment with UDCA and immunosuppressive therapy using prednisolone or budesonide, followed by the addition of antimetabolites such as mycophenolic acid or azathioprine [141]. A major clinical challenge is determining whether AIH or PBC is the dominant disease component when evaluating treatment response. If changes in liver enzymes in response to anti-cholestatic therapy and serum IgG levels are inconclusive, repeat liver biopsy may be required to identify the predominant pathology and guide second-line or third-line treatment decisions.

### Obeticholic acid (OCA)

The selective farnesoid X receptor (FXR) agonist OCA was the first drug receiving conditional approval in 2016 for second-line treatment of PBC [114]. Activation of FXR increases biliary bile acid excretion, reduces intestinal reabsorption, and suppresses bile acid synthesis [142]. In the POISE trial (*n* = 216), OCA 5–10 mg or 10 mg combined with UDCA achieved the primary endpoint in 46% and 47% of patients, respectively, compared with 10% in the placebo group; long-term extension data confirmed sustained biochemical benefit and improved transplant-free survival [114, 142, 143] (Tab. 2). The use of OCA was associated with higher rates of pruritus and lipid abnormalities, and later with increased hepatic decompensation risk in patients with compensated cirrhosis and portal hypertension, leading to a contraindication in this population. A subsequent phase 3b/4 trial (COBALT), required to confirm improved clinical outcomes, failed to demonstrate benefits over placebo and was terminated early, partly due to high drop-out rates in the placebo arm [144]. In June 2024, the EMA revoked conditional approval of OCA for PBC, and the drug is currently not available as second-line therapy in Europe. Access may still be possible through named patient programs in selected cases and triple therapy combining a PPAR agonist, FXR agonists, and UDCA may be considered as a potential future treatment option.

## Management of symptoms and extrahepatic manifestations

### Pruritus

Pruritus is a common and burdensome symptom in patients with PBC, yet clinician recognition and documentation could be improved. No drugs have been formally approved specifically for the treatment of pruritus in PBC, and first-line therapy with UDCA had not yet been demonstrated to significantly affect pruritus [10].

All patients suffering from pruritus should be advised on general supportive measures [10]. These include topical skin care with lipid-replenishing and moisturizing emollients (e.g., those containing urea), short lukewarm showers or baths, skin cooling measures, avoidance of circumstances increasing dryness and/or irritation of the skin, and the use of loose clothing made of natural fibers (cotton or linen) [31]. Fingernails should be shortened to avoid severe skin damage [145].

Phase 3 data indicate that selective PPAR agonists, particularly seladelpar, significantly improve moderate-to-severe pruritus, with 93% of patients achieving clinically meaningful improvement after 1 year [110, 112–114, 122–146].

Other current management options include bezafibrate, cholestyramine, rifampicin, naltrexone, sertraline, and ileal bile acid transporter (IBAT) inhibitors.

Among these, bezafibrate has shown improvement in pruritus severity [112–114, 132–149]. In PBC patients not already receiving combination therapy with a selective PPAR agonist, bezafibrate is thus recommended (*off-label *use) for treatment of pruritus [31].

Alternatively, the bile acid sequestrant and anion exchange resin cholestyramine may be used (up to 4 g 4 times daily) in mild pruritus, ensuring a 2–4‑h interval between administration and UDCA or other disease-modifying medication for PBC [31–61, 65–150]. Colesevelam, another anion exchange resin with a higher bile acid-binding affinity, was not superior to placebo in alleviating pruritus intensity in a randomized, placebo-controlled trial, despite an almost 50% reduction in systemic bile acid concentrations [151].

If cholestyramine or bezafibrate are ineffective or not tolerated, *off-label* therapy with the pregnane X receptor (PXR) agonist rifampicin (150–600 mg/day) may be considered [152–155]. Given the potential for drug-induced liver injury [156], close monitoring of liver transaminases (every 2–4 weeks after the begin of rifampicin) is mandatory at initiation and during rifampicin treatment. Rifampicin should be discontinued in the presence of signs of hepatocellular injury [157]. Rifampicin treatment may cause a harmless orange-red discoloration of body fluids, including urine, stool, and tears, of which patients should be advised.

Further *off-label* options include μ‑opioid receptor antagonists (naltrexone, 12.5–150 mg/day) or selective-serotonin receptor uptake inhibitors (sertraline, 50–100 mg/day) [145–158].

The IBAT inhibitor linerixibat (GSK2330672) at 40 mg twice daily has been shown to ameliorate moderate-to-severe pruritus compared with placebo (GLISTEN trial), as assessed by the worst itch numerical rating scale (WI-NRS ≥ 4) [159].

Non-pharmacological treatment options available at specialized centers include ultraviolet B therapy, extracorporeal liver support systems, and percutaneous or nasobiliary drainage [145].

### Fatigue

The diagnostic evaluation and management of fatigue in patients with PBC can follow a structured three-step ask-measure-treat algorithm [14]. This approach begins with systematic assessment of fatigue and associated symptoms, medication review, and contributing comorbidities, followed by grading fatigue severity (e.g. by using a visual analogue scale) (0 = no fatigue to 10 = worst fatigue imaginable) and performing targeted clinical and laboratory (blood count, serum iron parameters, glucose, electrolytes, kidney function, liver tests, ferritin, vitamin D, thyroid-stimulating hormone, exclude celiac disease) measurements. Management then focuses on identifying and treating underlying conditions (e.g. hypothyroidism, anemia), alleviating contributing symptoms such as sleep disturbances, depression, and autonomic dysfunction, promoting adaptive coping strategies, and maintaining an empathetic, patient-centered approach [160].

Currently, no pharmacological treatment can be recommended as no therapies are formally approved for the treatment of fatigue in PBC, and no drugs have demonstrated clear efficacy [13]. In the BEZURSO study, bezafibrate treatment was associated with some improvement in fatigue assessed using a simple patient-reported three-category scale (absent, intermittent, continuous) [112]. The selective PPAR agonists elafibranor and seladelpar have demonstrated some efficacy in improving moderate-to-severe fatigue in the respective phase 3 trials and open-label extension studies [110,111–114, 122–161].

Pilot studies suggest that nonpharmacological interventions, such as physical exercise, online body-mind programs, and morning bright-light therapy, may improve fatigue and sleep-wake disturbances in PBC [14–61, 65–165]; however, findings across these studies have been heterogeneous. In a randomized trial including 55 women with PBC and significant fatigue (PBC-HOPE), hypnosis and psychoeducation did not significantly reduce fatigue scores at 12 weeks compared with standard care, but both interventions improved patients’ subjective perceptions of fatigue without safety concerns [166].

### Associated diseases

In the management of patients with PBC, assessing potential comorbidities is essential, as they can influence disease progression, treatment tolerability and HRQoL.

Thyroid dysfunction is significantly more common in patients with PBC than in the general population [167–169]. Concomitant hypothyroidism should be considered, particularly in patients presenting with fatigue [31]. As PBC is an autoimmune disease, affected individuals are also at increased risk of developing autoimmune thyroid disorders, including Hashimoto’s thyroiditis and Graves’ disease [169]. The underlying mechanisms are not fully understood but may involve disrupted interactions between thyroid hormones and hepatic metabolism resulting from PBC-associated liver damage [170]. Early recognition and accurate diagnosis of thyroid dysfunction, followed by appropriate treatment, may improve symptoms and contribute to a better quality of life in patients with PBC.

In comparison with the general population, patients with PBC are at substantially increased risk of osteoporosis (relative risk, RR 2.79), bone fractures (odds ratio, OR 1.86) and postfracture mortality [171,172]. The prevalence of osteoporosis in PBC patients is around 30% [173], with higher rates seen in those with advanced liver disease, reaching up to 44% in individuals listed for liver transplantation [174]. Osteoporosis significantly increases the risk of fractures, with incidence rates ranging from 0–14% over a 2-year period in PBC patients. The prevalence of fractures is reported to be 10–20% [175], rising to 22% in patients on the transplantation waiting list [174]. Managing fractures is challenging especially in patients with advanced liver disease, with perioperative morbidity and mortality rates reaching 80% and 60% respectively in cirrhotic individuals undergoing urgent total hip replacement [176]. Despite the strong association between BMD and fracture risk, fewer than half of fractures are associated with reduced bone density, because most fractures occur at a T-score of ≥ −2.5 SD, which is above the threshold for a densitometric diagnosis of osteoporosis [177,178]. In addition to bone density, fracture risk is associated with a history of previous fractures, age, sex, body mass index, smoking, glucocorticoid treatment, and comorbidities. Timely diagnosis, effective modification of risk factors, and treatment of PBC-related osteoporosis are crucial for patient care. The Austrian Society of Bone and Mineral Research recommends the initial assessment of the 10-year fracture probability by FRAX score [179]. A version of FRAX® calibrated to the Austrian population is available (https://frax.shef.ac.uk/FRAX/tool.aspx?lang=de) [179]. Patients with low fracture risk do not have to be further evaluated by a bone densitometry using dual-energy X‑ray absorptiometry (DEXA), whereas those with an intermediate and high risk should be further evaluated by a DEXA. Treatment should be initiated depending on the risk if the intervention threshold is exceeded or if a prior fragility fracture is documented in the medical history [179]. At time of PBC diagnosis, serum levels of vitamin D, calcium, phosphorus, and parathyroid hormone (PTH) should be checked and reassessed annually [180].

While treatments for postmenopausal osteoporosis are available, managing osteoporosis in PBC is hindered by a lack of understanding of the disease-specific pathophysiology and limited research on potential treatments. According to current guidelines of the Austrian Society for Bone and Mineral Research, the prevention measures for the general population and those with low fracture risk of should include 1000 mg daily calcium, 800 IE daily vitamin D and 0.8–1.5 mg daily protein intake per kg body weight [179]. Osteoporosis treatment should be considered in patients with moderate and initiated in patients with high risk of osteopenic fractures [179]. Bisphosphonates (alendronate, risedronate, ibandronate, zoledronate) and denosumab are effective medications. Alternative options include hormone replacement therapy and raloxifene. Patients with high risk should be followed in specialized centers and treated with osteoanabolic therapy [179].

Sicca syndrome is common in patients with PBC (in 10–25%), often presenting with symptoms such as dry eyes and/or dry mouth [181–183]. Additional symptoms may include difficulty swallowing and vaginal dryness. These symptoms can be seen in patients with and without an association with Sjögren’s disease [184]. Association of PBC and Sjögren’s disease has been reported in 19–31% of PBC patients [9]. Treatment with artificial tears and saliva can provide symptom relief. In cases where symptoms are persistent pilocarpine can be prescribed [10]. Patients with severe xerostomia should receive oral hygiene advice to prevent dental caries and be monitored for the development of oral candidiasis. Vaginal moisturisers can provide relief, while the use of estrogen creams should be managed by gynecologists. Consultation with a rheumatologist is recommended for the diagnosis and management of Sjögren’s disease, particularly in patients with persistent symptoms, as new therapeutic options continue to emerge [10].

Hypercholesterolemia associated with PBC affecting up to 80% of patients can lead to xanthomas and/or xanthelasmas and is largely driven by lipoprotein X, which inhibits LDL oxidation and may mitigate atherosclerotic risk [185,186].

Accordingly, most studies do not show increased atherosclerosis or cardiovascular risk in PBC patients without additional risk factors, with arterial hypertension being the key determinant of risk [187–189]. In a large multicenter retrospective cohort study of 14,598 patients with PBC (2012–2024), presented at The Liver Meeting 2025, hyperlipidemia was associated with significantly higher rates of major adverse cardiovascular events and metabolic comorbidities, including ischemic heart disease, heart failure, stroke, peripheral arterial disease, hypertension, and type 2 diabetes, challenging the longstanding notion that hyperlipidemia in PBC is benign [190].

Furthermore, emerging evidence suggests that disturbances in lipid metabolism may be associated with fibrosis severity and liver-related outcomes and may therefore have prognostic value in PBC, although further validation is needed [191,192].

Lipid assessment is thus recommended in all patients with PBC and decision on initiating treatment for dyslipidemia should be based on the risk for the development of major cardiovascular events calculated by the appropriate scores (e.g. SCORE or SCORE-2).

## Liver transplantation in PBC

Despite an increasing prevalence of PBC, its relative contribution as an indication for liver transplantation has declined over recent decades. Data from Europe show a decrease from 20% to about 5% of liver transplantations, with a slight increase in age (from 54 years, IQR 47–59 years to 56 years, IQR 48–62 years) and proportion of male patients (from 11% to 15%). This might be largely due to earlier diagnosis and the widespread implementation and early initiation of UDCA therapy. Currently, PBC accounts for approximately 3.6–9.7% of adult liver transplantations [13–61, 65–193,194].

The criteria for liver transplantation in patients with PBC are broadly aligned with those for other chronic liver diseases; however, patients with PBC on the transplantation waiting list exhibit a comparatively higher mortality [195–198]. A key limitation is that advanced disease in PBC may be underestimated by MELD or MELD-Na scores, as these do not adequately capture cholestatic complications such as severe jaundice or manifestations of portal hypertension [197]. Furthermore, the occurrence of two or more episodes of hepatic decompensation is associated with a markedly increased 6‑month mortality (odds ratio 6.4) [199]. These considerations underscore the importance of timely referral and listing for transplantation [198–200].

Referral for transplantation evaluation is recommended in patients with evidence of disease progression despite optimal medical therapy. Key indicators include a progressive rise in serum bilirubin (≥ 3 mg/dL) or the development of cirrhosis-related complications [201,202]. Intractable pruritus refractory to medical therapy represents an additional indication for referral, whereas fatigue alone should not prompt transplantation evaluation [201,202].

Listing for liver transplantation should follow established guideline recommendations. Liver transplantation in PBC is associated with favorable outcomes, with 5‑year survival rates of 82–92% exceeding those observed for many other indications. Nevertheless, certain symptoms, particularly fatigue, may persist after transplantation. Long-term management should also address symptoms (e.g. fatigue) and comorbidities (e.g. osteoporosis, autoimmune diseases).

Recurrent PBC occurs in a substantial proportion of patients following transplantation, with cumulative incidences of up to 22% at 5 years and up to 50% at 15 years; the median time to recurrence is approximately 4.4 years [203,204]. Recurrent primary biliary cholangitis negatively impacts graft and overall patient survival after liver transplantation [205]. Identified risk factors for recurrence include younger age at diagnosis or transplantation, the use of tacrolimus-based immunosuppression, and the presence of post-transplant biochemical cholestasis [203]. Histological assessment remains the diagnostic gold standard for recurrent PBC [195]. Histological features of recurrent PBC include the presence of florid duct lesions or destructive lymphocytic cholangitis with significant portal infiltrate in the absence of endothelialitis [205].

In patients with established or suspected recurrent PBC, UDCA can be safely administered and is often associated with improvement in liver biochemistry. Importantly, emerging evidence indicates that pre-emptive UDCA therapy (10–15 mg/kg/day), initiated immediately after transplantation, is associated with a reduced risk of disease recurrence, graft loss, and mortality. These findings support the routine use of pre-emptive UDCA in all patients undergoing transplantation for PBC [206–208]. In histologically confirmed recurrent PBC, PPAR agonists may be considered; however, evidence supporting this strategy remains limited, and transplant-specific safety concerns including potential drug-drug interactions should be carefully evaluated [56].

## PBC and pregnancy

Pregnancy in biochemically well-controlled and compensated PBC patients is not associated with adverse maternal of fetal outcome [10]; however, pregnant women with PBC should be closely monitored by obstetricians in cooperation with an experienced hepatologist, particularly when ACLD is present. Postpartum cholestatic flares have been described, thus, narrow follow-up in the postpartum period seems reasonable [10].

The administration of UDCA should be continued as it appears to be safe during pregnancy when given at recommended doses [209,210]. There is no evidence to indicate that UDCA is teratogenic in humans. While there is a paucity of data from controlled studies documenting its use in pregnancy and breastfeeding, it is deemed safe for administration before and throughout the first trimester, as well as during subsequent stages of pregnancy [10]. Although guideline recommendations are lacking due to limited data, available case reports and studies indicate that UDCA does not significantly accumulate in breast milk and appears safe during breastfeeding, with negligible infant exposure and no reported adverse effects [209–211,212].

Management of pruritus is an important aspect of care and may necessitate specialist advice, with cholestyramine or rifampicin (3^rd^ trimester) as potential *off label* options [209–213]. The 2023 EASL Clinical Practice Guidelines on liver diseases in pregnancy state that fibrates may be considered after the first trimester as add-on therapy to UDCA, provided the treating team judges that the potential benefits outweigh the associated risks. In this context, case reports have described the safe use of bezafibrate during pregnancy [214]. For the selective PPAR agonists elafibranor and seladelpar, no data during pregnancy or breastfeeding are available, as they have not been investigated in this context; they are therefore contraindicated during gestation and lactation [64].

## Data Availability

All data supporting the findings of this work are included in the article.

## Abbreviations

A1-D2: Strength of recommendation and certainty of evidence according to the GRADE framework
ABCB4: ATP-binding cassette subfamily B member 4
ABCB11: ATP-binding cassette subfamily B member 11
ACA: Anti-centromere antibody
ACLD: Advanced chronic liver disease
AIH: Autoimmune hepatitis
ALP: Alkaline phosphatase
ALT: Alanine aminotransferase
AMA: Anti-mitochondrial antibody
ANA: Anti-nuclear antibody
Anti-HK1: Anti-hexokinase 1
Anti-KL12: Anti-kelch-like 12
AST: Aspartate aminotransferase
ATP8B1: ATPase phospholipid transporting 8B1
BSEP: Bile salt export pump
BW: Body weight
CK: Creatinine kinase
CKD: Chronic kidney disease
CSPH: Clinically significant portal hypertension
DEXA: Dual-energy X‑ray absorptiometry
ELF: Enhanced liver fibrosis
ELISA: Enzyme-linked immunosorbent assay
EMA: European Medicines Agency
EU-SMPC: EU Summary of Product Characteristics
EUS: Endoscopic ultrasound
FIC1: Familial intrahepatic cholestasis 1
FIS: Fatigue impact scale
FSS: Fatigue severity score
FXR: Farnesoid X receptor
GFR: Glomerular filtration rate
GGT: Gamma-glutamyltransferase
GR: Glucocorticoid receptor
GRADE: Grading of recommendations, assessment, development, and evaluations
HCC: Hepatocellular carcinoma
HRQoL: Health-related quality of life
HVPG: Hepatic venous pressure gradient
IBAT: Ileal bile acid transporter
Ig: Immunoglobulin
IQR: Interquartile range
LC‑1: Liver cytosol type 1 antibody
LKM‑1: Liver kidney microsomal antibody type 1
LLN: Lower limit of normal
LSM: Liver stiffness measurement
MASLD: Metabolic dysfunction-associated steatotic liver disease
MDR3: Multidrug resistance protein 3
MRCP: Magnetic resonance cholangiopancreatography
NADPH: Nicotinamide adenine dinucleotide phosphate
NOX: NADPH oxidase
NRS: Numerical rating scale
OCA: Obeticholic acid
ÖGGH: Austrian Society for Gastroenterology and Hepatology
PBC: Primary biliary cholangitis
PPAR: Peroxisome proliferator-activated receptor
PRO: Patient reported outcome
PSC: Primary sclerosing cholangitis
PTH: Parathyroid hormone
PXR: Pregnane X receptor
SLA/LP: Soluble liver antigen/liver pancreas antibody
SMA: Smooth muscle antibodies
SSC: Secondary sclerosing cholangitis
SSM: Spleen stiffness measurement
TSH: Thyroid stimulating hormone
UDCA: Ursodeoxycholic acid
ULN: Upper limit of normal
VAS: Visual analogue scale
VCTE: Vibration-controlled transient elastography
